# Quantitative assessment of outer retinal folds on enface optical coherence tomography after vitrectomy for rhegmatogenous retinal detachment

**DOI:** 10.1038/s41598-019-38751-z

**Published:** 2019-02-20

**Authors:** H. Fukuyama, H. Yagiri, T. Araki, H. Iwami, Y. Yoshida, H. Ishikawa, N. Kimura, K. Kakusho, T. Okadome, F. Gomi

**Affiliations:** 1 Department of Ophthalmology, Hyogo College of Medicine, Nishinomiya, Japan; 2 School of Science and Technology, Kwansei Gakuin University, Sanda, Japan

## Abstract

We aimed to investigate the possible causes of metamorphopsia for rhegmatogenous retinal detachment (RRD) based on findings of enface optical coherence tomography (OCT). The study was a retrospective, consecutive case series of 33 eyes with macula-off RRD. Metamorphopsia was measured by M-CHARTS and enface OCT images covering a macular area of 6 × 6 mm square obtained at 1, 3, and 6 months postoperatively. Within the outer retinal slabs of enface OCT, multiple lines that looked like folds were delineated in all eyes at month 1, and we succeeded in extracting images of folds by subtracting retinal vessels. We calculated their density by an image-processing technique. The mean M-CHARTS scores were 0.62 ± 0.47 at month 1 and 0.30 ± 0.29 at month 6 (P < 0.001). The fold density was 8.3 ± 4.2 at month 1 and 6.1 ± 3.1 at month 6 (P = 0.0044). The M-CHARTS scores at 6 months were significantly associated with the fold density at 1 month (r = 0.515, P = 0.002). In conclusion, enface OCT visualized the outer retinal folds in eyes that had undergone successful RRD surgery, and a larger number of folds was related to the remaining metamorphopsia.

## Introduction

Rhegmatogenous retinal detachment (RRD) is caused by one or more breaks in the retina, resulting in fluid from the vitreous cavity accumulating between the neurosensory retina and the retinal pigment epithelium. Surgery is the only effective treatment for RRD and the final anatomical success rate is almost 100%^1–5^, but it does not always succeed in restoring visual function^6–9^.

Metamorphopsia is one of the most common postoperative complaints, and the incidence is much higher in macula-detached (macula-off) RRD cases^10–14^. Previous studies have indicated the relationship between lesser improvement of visual acuity and morphological abnormalities of the retina on the basis of cross-sectional optical coherence tomography (OCT) findings after RRD repair, but OCT abnormalities relating to postoperative metamorphopsia have not been fully investigated^12,15,16^. Dell’Omo *et al*. suggested that outer retinal folds seen on cross-sectional OCT after repair of RRD could be associated with persistent metamorphopsia^17,18^. Recent advances in OCT technology have made possible the reconstruction of enface images from OCT volume scans. Enface OCT can delineate the localization and the spatial extent of abnormal regions at arbitrary depth and images can be easily registered with data acquired from other imaging modalities. Advantages of enface OCT imaging of outer retinal pathologies have been demonstrated in the management of age-related macular degeneration^19^.

The purpose of this study was to assess microstructural changes within the outer retina on enface OCT images after RRD repair and to investigate their relationship to metamorphopsia.

## Results

Forty-six eyes of 46 consecutive patients showed anatomical reattachment of the retina after surgery. Of these, 13 eyes—preexisting macular condition (1 eye), PVR Grade C (2 eyes), RRD with macular hole (2 eyes), second surgery for re-RRD (1 eye) and for epiretinal membrane (1 eye), followed up for less than 6 months (6 eyes) — were excluded. Thus 33 eyes of 33 patients with RRD were included in the study.

**Figure 1:**
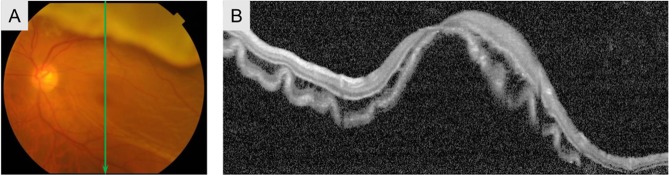
Findings for a 53-year-old man with rhegmatogenous retinal detachment repair. Color fundus camera before surgery showed a corrugated fundus appearance (**A**), and B-scan OCT showed undulation of the outer retina (**B**).

Table 1 shows preoperative clinical characteristics. Nineteen were men and 14 were women, and the mean age was 55.4 years. All eyes had a macula-off RD (defined as an RD involving the fovea) with an irregular outer surface (Fig. 1A,B). The mean interval between symptom onset and surgery was 12.3 ± 17.4 days (range, 2–90 days).

**Table 1:** Baseline characteristics.

Number of patients (eyes)	33 (33)
Age (yrs)	55.4 ± 10.4
Gender (male/female)	19/14
Preoperative visual acuity (logMAR)	0.76 ± 0.74
Number of breaks	1.4 ± 0.6
Location of RD (superior/inferior)	31/2
Extent of RD (hours)	5.7 ± 2.0
Lens status (phakic/pseudphakic)	33/0
Duration of symptoms (days)	12.3 ± 17.4

BCVA = best-corrected visual acuity; logMAR = logarithm of the minimum angle of resolution; RD = retinal detachment.

### Time course of changes in BCVA and metamorphopsia after RRD Surgery

The preoperative mean BCVA was 0.76 ± 0.74 logarithm of the minimum angle of resolution (logMAR; range, −0.079–2.3). The mean logMAR BCVA postoperative 1, 3, and 6 months after vitrectomy were 0.12 ± 0.18, 0.074 ± 0.12, and 0.065 ± 0.20 respectively. Postoperative BCVA improved significantly for all examination time periods (P < 0.001), and BCVA at 3 and 6 months improved significantly over BCVA at 1 month (P = 0.028 and P = 0.049 respectively).

The vertical metamorphopsia (MV) scores at 1, 3, 6 months postoperatively were 0.68 ± 0.47, 0.46 ± 0.35, and 0.34 ± 0.31 respectively, and the horizontal metamorphopsia (MH) scores were 0.56 ± 0.54, 0.37 ± 0.45, and 0.26 ± 0.36 respectively. Compared to the scores at 1 month, the MV and MH scores reduced both at 3 months (P < 0.001 and P = 0.064, respectively) and at 6 months (P < 0.001 and P < 0.001, respectively). The average of MV and MH (mean M-CHARTS scores) were 0.62 ± 0.47, 0.42 ± 0.34, and 0.30 ± 0.29 at 1, 3, 6 months respectively, and the scores at 3 and 6 months improved significantly over the score at 1 month. (P = 0.002 and P < 0.001 respectively).

### Enface and cross-sectional OCT imaging of folds

**Figure 2:**
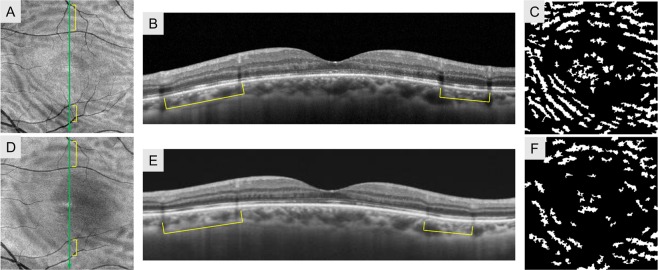
Finding for the same patient as shown in Fig. 1 with rhegmatogenous retinal detachment repair. Enface OCT of the outer retinal slab at 1 month showed several folds with dull hyporeflective lines (**A**). Corresponding lesions on B-scan OCT at 1 month after vitrectomy showed disrupted outer retinal bands (**B**). A binary image of enface OCT (**C**) showed extracted folds. The density of folds was 19.72 respectively. Folds on enface OCT became unclear at 6 months postoperatively (**D**). In the B scan OCT images at 6 months, disruption of the outer retinal bands was less apparent than at 1 month (**E**). The density of folds in the binary images of enface OCT decreased to 11.73 respectively (**F**).

**Figure 3:**
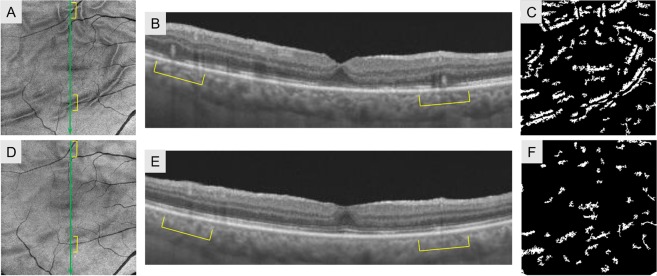
Findings for the right eye of a 58-year-old man with rhegmatogenous retinal detachment repair. Enface OCT of the outer retinal slab at 1 month postoperatively showed several folds with sharp outlines (**A**). The corresponding region on cross-sectional OCT showed elevated outer retinal bands (**B**). Binary images of enface OCT showed folds. The density of folds was 15.71 respectively (**C**). At 6 months, the folds in the enface OCT images were indistinct (**D**), and the elevation of the outer retinal bands on B-scan OCT regressed with less reflectivity (**E**). The total number and density of folds in the binary images of enface OCT at 6 months decreased to 7.09 respectively (**F**).

Enface OCT of the outer retinal slab at 1 month after surgery showed one or more folds in all cases (100%). There were two types of folds from the appearance: a dull hyporeflective line (Fig. 2A) and a sharp hyperreflective line with hyporeflective lines on both sides (Fig. 3A). The former pattern was seen in all eyes (100%) and the latter one in eight eyes (24.2%). In the cross-sectional OCT images of the hyporeflective lines, intermittent disruption of outer retinal bands (external limiting membrane, ellipsoid zone, and interdigitation zone) was seen (Fig. 2B). The corresponding regions of sharp folds showed elevation of the outer retinal bands within the hyporeflective outer retinal bands (Fig. 3B). These findings were apparent on both enface and cross-sectional OCT at 1 month after surgery and they gradually resolved through months 3 to 6 postoperatively (Figs 2 and 3). The sharp folds in enface OCT transformed into dull folds during the time course. The binary image reproduced those folds and traced their time-dependent changes (Figs 2C,F and 3C,F).

### Time course of changes in the parameters of the folds

**Figure 4:**
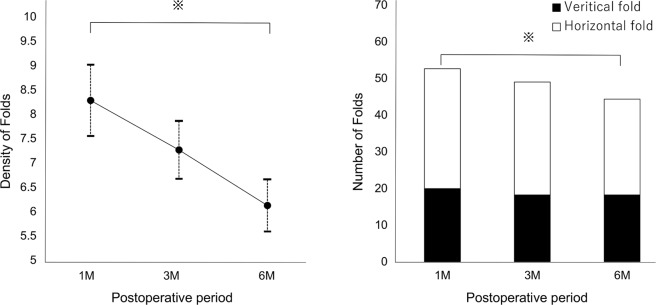
Time course of density of folds (**A**) and number of folds (**B**) on enface OCT at 1, 3, and 6 months after vitrectomy for macula-off rhegmatogenous retinal detachment repair. Vertical bars represent mean ± standard error. ^*^P < 0.05, ^†^P < 0.01.

Table 2 shows the time course of the parameters of the folds. The density of folds decreased significantly from month 1 to month 6 (P = 0.004) (Fig. 4A).

**Table 2:** Time course of changes in parameters of folds.

	Postoperative 1 month	Postoperative 3 months	Postoperative 6 months	P
Density (%)	8.28 ± 4.21	7.26 ± 3.31	6.12 ± 3.08	0.004^†^
Number				
Total	53.2 ± 17.1	48.9 ± 15.8	44.3 ± 16.0	0.030^*^
Vertical	19.9 ± 8.7	18.2 ± 7.9	18.2 ± 8.2	0.75
Horizontal	33.2 ± 16.7	26.8 ± 17.6	24.9 ± 13.7	0.006^†^
Length (pixels)	27.5 ± 3.2	27.0 ± 2.9	26.4 ± 2.7	0.41
Width (pixels)	13.0 ± 1.9	13.7 ± 1.9	13.8 ± 2.2	0.21

The total number of folds and the number of horizontal folds decreased significantly from month 1 to month 6 (P = 0.030 and P = 0.006 respectively). The numbers of vertical folds were not significantly different. The number of horizontal folds was significantly larger than that of vertical folds at all postoperative points (P < 0.001, P < 0.001, and P = 0.002 respectively) (Fig. 4B).

The mean lengths of folds and the mean widths of folds were not significantly different (P = 0.41 and P = 0.21 respectively).

### Relationship between metamorphopsia and folds

The correlations between the density of folds and the mean M-CHARTS scores are shown in Table 3. The density of folds correlated significantly with the mean M-CHARTS scores at 1 month. (r = 0.419, P = 0.015). Moreover, the density of folds at 1 month correlated significantly with the M-CHARTS scores at 6 months (r = 0.515, P = 0.002). Similarly, the number of folds at 1 month correlated significantly with the mean M-CHARTS scores at 1 month (r = 0.435, P = 0.011), and with the mean M-CHARTS score at 6 months (r = 0.532, P = 0.002). The mean lengths and width of folds were not correlated with postoperative mean M-CHARTS score at any postoperative examination time point.

**Table 3:** Correlations Between mean M-CHARTS scores and the density of folds after surgery.

	M-CHARTS scores 1 month		M-CHARTS scores 3 months		M-CHART scores 6 months	
	r	P	R	P	r	P
Density of folds 1 month	0.419	0.015*	0.325	0.065	0.515	0.002^†^
Density of folds 3 months			0.042	0.82	0.162	0.38
Density of folds 6 months					0.283	0.11

*Significant at P < 0.05 (Spearman’s correlation coefficient by rank test).

^†^Significant at P < 0.01 (Spearman’s correlation coefficient by rank test).

## Discussion

We aimed to investigate morphological changes as evaluated by enface OCT, which can cause persistent metamorphopsia after surgery for macula-off RRD. Multiple lines that appeared as folds were frequently apparent in postoperative enface OCT images of the outer retinal slab. Then we proceeded to extract folds from whole images and analyzed their time-dependent changes and the relationship with subjective symptoms of metamorphopsia.

The advantages of enface OCT imaging have been reported. Enface OCT produces transverse images of retinal layers at any specified depth. In this study, enface OCT delineated folds clearly with the information of number, length and width, and direction. Imaging analysis combined with OCTA enabled us to extract folds from whole image including blood vessels or other artifacts.

As to the imaging analysis, binalization methods are widely used. However, binalization process may cause to lose important but subtle changes or to incorporate artifacts or noises. In this study, we did efforts to extract pure folds effectively combining several image analyzing procedures^20^. That allowed us the quantitative analysis of the characteristics of postoperative folds of macula-off RRD. We believe that these procedures can be applied to any retinal disease.

Findings of retinal folds on B scan OCT have been reported by Dell’Omo *et al*.^17,18^. They found the upfoldings of the outer retinal layers in eyes with vitrectomy for RRD and called them as outer retinal folds. In the present study, we found two types of folds as to appearance in enface OCT: a sharp hyperreflective line with hyporeflective lines on both sides, and a dull hyporeflective line, the former type of folds was detected in 24% of eyes and the latter type in all eyes. In corresponding OCT B-scan imaging, there were also two types of changes: partial disruption of outer retinal bands with and without upfolding. During follow-up, the sharp folds became dull with less reflectivity on enface OCT, and the corresponding B scan images showed flattening of the outer retinal bands with disrupted reflectivity. Therefore, we believe that the two different kinds of folds have same origin: the severity of the preoperative outer retinal undulations. Those consequent changes were similar to the findings of outer retinal folds observed on B scan image by Dell’Omo *et al*.^17,18^.

In preoperative fundus examination, detached retina showed a crepe pattern, and B scan OCT depicted the undulated outer surface of the detached retina with swollen outer nuclear and plexiform layers (Fig. 1). The postoperative folds in enface OCT were seen at the areas of undulations of the detached retina (Fig. 2). These findings support the hypothesis of previous reports that preoperative undulations of the outer retina profile result in postoperative outer retinal folds although these folds were undetectable ophthalscopically^17,18^. The outer retinal folds seen as sharp folds in enface OCT gradually decreased in height and became dull folds, but at 6 months after vitrectomy, they were still visible in enface OCT. Thus, both types of folds originated from preoperative outer retinal corrugation.

Associations between metamorphopsia and retinal morphological changes on B-scan OCT image after macula-off RRD repair have been reported^12,15,16,21^. The presence of SRF, disruption of external limiting membrane lines^21^, disruption of the interdigitation zone and the increase of external limiting membrane-retinal pigment epithelium thickness are possible factors of postoperative metamorphopsia^15^. Okuda *et al*. reported that restoration of both the ellipsoid zone and interdigitation zone bands appeared to be an important factor in the reduction of metamorphopsia after successful vitrectomy for macula-off RRD^16^. Metamorphopsia, however, can be due to lateral photoreceptor displacement in any direction, and hence evaluation of only one or a few slices of cross-sectional OCT images might be insufficient to assess metamorphopsia.

In the course of this study, we hypothesized that preoperative outer retinal corrugation caused subclinical but persistent postoperative outer retinal folds and that those folds could be associated with postoperative metamorphopsia in eyes with macula-off RD. Then, we analyzed various parameters of folds including the number, length, and direction of each fold (Table 3). The number and density of folds at I month were correlated with the M-CHARTS scores at 6 months. The results suggest that outer retinal changes over a wide area surrounding the macula region influence metamorphopsia and folds at the outer retinal layer on enface OCT in the early postoperative period is a predictor of persistent metamorphopsia.

Interestingly, the horizontal folds were significantly more numerous than the vertical folds, and the vertical M-CHART scores were consistently higher than the horizontal scores. These results accord with the hypothesis presented in previous reports that photoreceptor and/or its outer segments dislocated in the horizontal direction can cause an increase in the distortion of the vertical line^22^. During the time course, the horizontal folds reduced more significantly than the vertical folds. Shiragami *et al*. observed downward movement of the retina after vitrectomy for RRD^23^. They reported that the retina might move slightly upward to the original position. Such a vertical shift of the retina might reduce the horizontal folds more than the vertical folds, although both vertical and horizontal metamorphopsia reduced significantly during the time course of the study. Further investigations into the relationship between the direction of folds and metamorphopsia are requisite.

There were several limitations in the present study. First, limitations stemmed from OCT measurements. Folds in the enface image were depicted differently when the setting was changed from the default “outer retina” slab. Second, image analysis process could affect the results. Minor unexpected artifacts in the enface OCT images might have been yet included as folds after binalization. On the contrary, ambiguous retinal folds could not be correctly distinguished. Third, there were several surgeons and surgical procedures, but Peirett *et al*. reported that the use of adjuvants and variations in postoperative position did not change the risk of the development of outer retina folds, or the dropping out of E-Z lines after RRD. Fourth, we did not analyze the relationship between the distribution of postoperative folds and preoperative RRD characteristics such as extent, height, duration, and the location of retinal breaks. Other limitations were a relatively small sample size, the retrospective character of the study, and shorter follow-up periods. Nevertheless, the present study indicates that usefulness of enface OCT evaluation and our imaging procedures to know the presence of both apparent and subsided outer retinal folds after vitrectomy for RRD and to perform quantitative analysis.

In conclusion, enface OCT detected outer retinal microstructural changes as folds after macula-off RRD repair. These folds might be responsible for persistent metamorphopsia in patients with anatomically successful RRD repair.

## Methods

The Institutional Review Boards of Hyogo College of Medicine and Kwansei Gakuin University approved this study, which followed the guidelines of the Declaration of Helsinki. We performed a retrospective study of consecutive patients with RRD who underwent primary vitrectomy at the Hyogo College of Medicine between October 2016 and August 2017. Written informed consent was obtained from each patient. Vitrectomies were performed by five experienced surgeons (H.F., T.A., Y.Y., H.I. and N.K.).

Exclusion criteria included preexisting macular conditions (e.g., age-related macular degeneration, vascular occlusive disease, and diabetic retinopathy) and complicated vitreoretinal diseases (proliferative vitreoretinopathy Grade C and RD with macular hole) that required additional surgery (re-RRD, cataract, epiretinal membrane) during the follow-up period. Also excluded were patients who were followed up for less than 6 months.

All patients underwent comprehensive ophthalmic examinations, including measurements of BCVA and intraocular pressure, degree of metamorphopsia, fundus examination, and OCT examination 1, 3, and 6 months after vitrectomy. BCVA was measured by Landolt C chart. The degree of metamorphopsia was quantified by M-CHARTS (Inami, Tokyo). The M-CHARTS consisted of 19 dotted lines with dot intervals between visual angles of 0.2 and 2.0 degrees. When the patient recognized a dotted line as straight, the visual angle that separated the dots was considered to represent his or her metamorphopsia score. Scores were obtained for vertical and horizontal tests separately, and their mean values were used for further data analysis. The examination was performed at 30 cm, and the refraction of the eye was corrected exactly for this distance. The examinations were repeated 3 times for each subject to evaluate the reproducibility of the test.

### Surgical techniques

Local anesthesia was induced by subtenon injection of 2% lidocaine. Phacoemulsification was performed in eyes with cataract of grade 2 nuclear sclerosis or cortical opacity. Twenty-five-gauge pars plana vitrectomy was performed in all cases using a vitrectomy system (Constellation; Alcon Labs, Fort Worth, TX). No scleral buckling procedure was performed on any of the patients enrolled in this study. Central and peripheral vitrectomy with release of vitreous tractions, shaving of the vitreous base, and cutting of the flaps of the breaks were performed using a non-contact wide-viewing system in all cases. Perfluorocarbon liquid was not used in any case. Particular care was paid to maximal drainage of the SRF during air-fluid exchange. Twenty percent sulfurhexafluoride gas was used as internal tamponade in all cases. Prone position was required in all patients immediately after surgery for 2–3 days postoperatively.

### Imaging

OCT images were obtained by swept-source (SS)-OCT (DRI-OCT Triton, Topcon, Tokyo). Cross-sectional OCT images were centered on the fovea, and enface OCT and OCTA images covered an area 6 × 6 mm square including the macula. We used preset segmentation slabs on enface OCT and OCTA and assessed outer retinal pathologies in the outer retinal slab (70.2 µm below the border between the inner plexiform layer (IPL) and the inner nuclear layer (INL) to the Bruch membrane). Multiple fold-like changes were observed, then we proceeded to extract them for the quantitative assessment. We used the term “fold” for these lines in this manuscript.

### Extraction of folds

**Figure 5:**
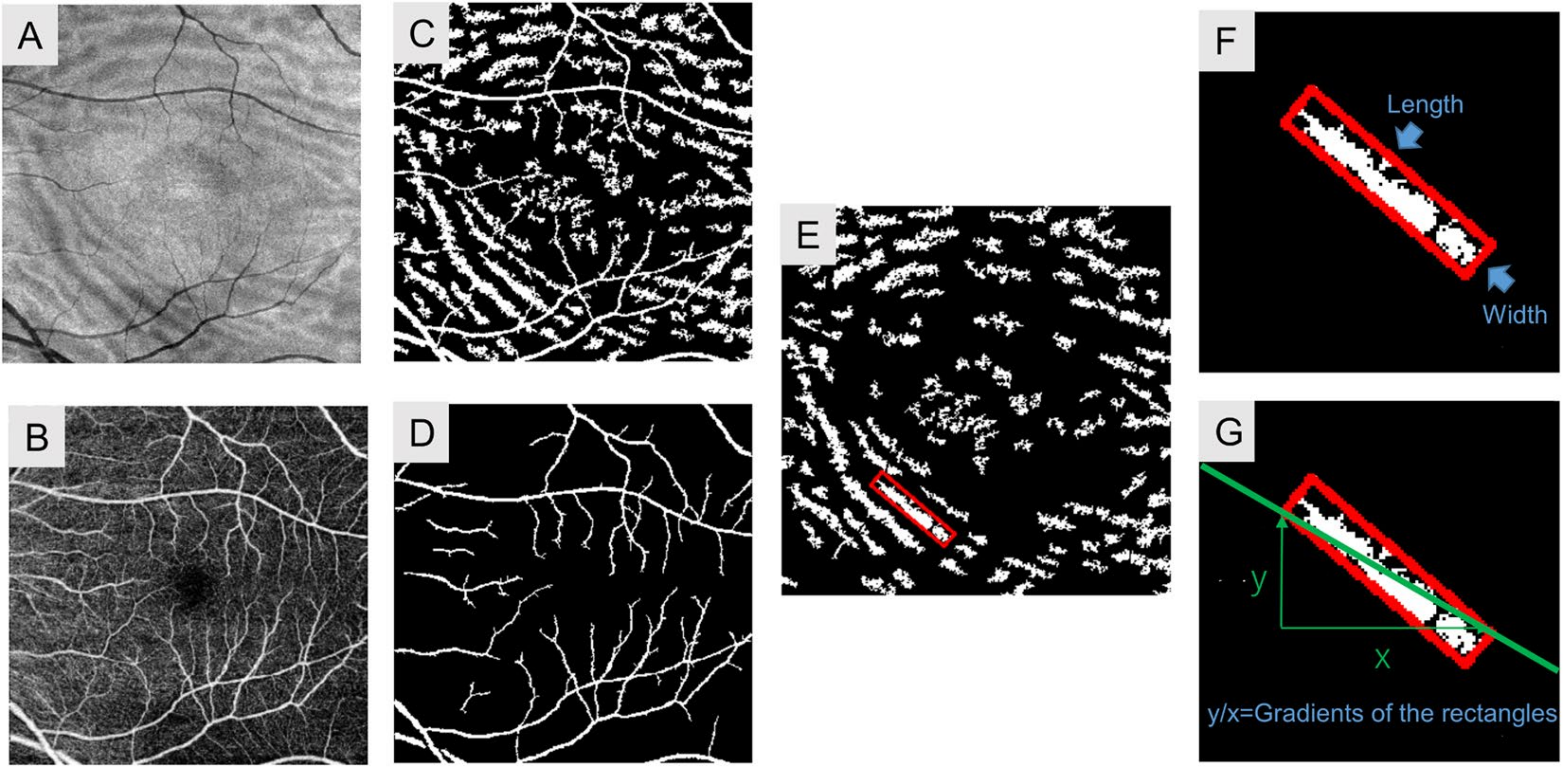
Method of enface OCT Image analysis of macula-off rhegmatogenous retinal detachment. Enface OCT of the outer retinal slab (**A**) and OCT angiography of the superficial retinal slab (**B**) were converted to binary images by the adaptive thresholding method. The binarized enface OCT image of the outer retinal slab showed fold-like changes, and an artifact of the blood vessels (**C**) and the binarized enface OCTA image of the superficial retinal slab showed blood vessels (**D**). The image of binarized enface OCT minus the image of binarized OCTA yielded an image that shows only the fold-like changes (**E**). Each fold was identified by its contours, and a bounding rectangle was drawn circumscribing each fold. The length of the long side of the rectangle was defined as the length of the fold, and that of the short side was defined the width (**F**). The gradient of the fold was defined as the slope of the diagonal of a rectangle (**G**).

Enface OCT images of the outer retinal slab and enface OCTA images of the superficial retinal slab (2.6 μm below the inner limited membrane to 15.6 μm below the border between IPL and INL) were used in subsequent image extraction analysis (Fig. 5A,B). The OCT images were saved in TIFF format (320 × 320 pixels) and analyzed by means of OpenCV software. We used the total variation filtering method to eliminate noise in the gray-scale images. Noise removal in the gray-scale images was accomplished by minimizing the following equation:

$$F(u)=\sum _{i,j}|\nabla {u}_{i,j}|+\frac{1}{2\lambda }{\sum _{i,j}\Vert {u}_{i,j}-{g}_{i,j}\Vert }^{2}$$

where g = input image, u = output image, ∇u = differential value of original image, i and j are pixel position, and λ = positive constant. The first term on the right side is a term for eliminating noise and the second term on the right side is a term for preventing the original image and the output image from changing. As λ increases, the difference between the output image and the original image increases. If λ = 0, the outcome is the original image. The Chambolle algorithm was used to minimize total variation in gray-scale images^24^. The filter smooths the gradient on the image while suppressing changes in the entire image. Therefore, it is possible to eliminate noise while leaving an edge like a boundary between folds.

We proceeded to convert these output images to binary images by the adaptive thresholding method. This method applies different thresholds for different regions of an image. The thresholds were defined based on the pixel values of a target pixel and the average pixel values of pixels peripheral to the target pixel. In this study, the thresholds were set to the average intensity within the 35 × 35 pix window plus 6 which provided appropriate image quality of blood vessels in the OCT angiography and folds with the artifacts of the blood vessels in enface OCT images (Fig. 5C,D). Finally, subtraction of both images enabled to delineate folds as white pixels. From the binary images, we removed those blobs that consisted of less than 50 white pixels in order to remove noise (Fig. 5E).

### Quantitative assessment

The OpenCV findContours function was used to determine the contour of each fold. Then we drew a bounding rectangle by the minAreaRect function. The coordinates of all four corners of a rectangle were outputted as X Y values based on the bottom left corner with positive X increasing to the right and positive Y increasing to the top. The lengths of the long and narrow sides of the rectangle of a given fold were defined as the length and width of the fold respectively (Fig. 5F). The pixel ratio of folds within the whole image was defined as the density of folds. The gradient of fold was defined as the slope of the diagonal of a rectangle (△Y/△X). We calculated the number of folds, the mean length and width of each fold (in pixels), and the densities of folds (%) at 1, 3, and 6 months after surgery. Based on the gradients of the rectangles of the various folds, we classified folds as “vertical” (over 1 and under −1) or “horizontal” (over −1 and under 1) and evaluated the numbers over the course of the study (Fig. 5G).

### Statistical analysis

The decimal BCVA was converted to the logMAR units for statistical analysis. Based on previous reports, counting fingers and hand motion were set to 2.0 and 2.30 logMAR units respectively^25^. All statistical analyses were performed by means of EZR software (Saitama Medical Center and Jichi Medical University)^26^. Data were expressed as mean ± SD. The Wilcoxon signed-rank test was used to compare the BCVA and M-CHARTS scores before and after treatment. The Mann-Whitney U test was used for comparison between the MV and the MH scores, and between the numbers of vertical and horizontal folds. The Friedman test was used to clarify changes in visual function and the parameters of the folds. Upon detecting a significant difference, we conducted the Bonferroni post hoc test for multiple comparisons to determine the time point that marked a significant difference. Correlations between mean M-CHARTS scores and the parameters of the folds were determined by Spearman rank correlation coefficient. P < 0.05 was considered statistically significant.
